# Porous bismuth-based liquid metal as multifunctional material

**DOI:** 10.1016/j.isci.2026.116028

**Published:** 2026-05-22

**Authors:** Ju Wang, Yan Wang, Yunlong Bai, Jingyi Li, Jie Zhang, Minghui Guo, Zhongshan Deng, Yong Zhang, Jinpeng Zhang, Wei Rao, Jing Liu

**Affiliations:** 1 State Key Laboratory of Cryogenic Science and Technology, Technical Institute of Physics and Chemistry, Chinese Academy of Sciences, Beijing 100190, China; 2 School of Future Technology, University of Chinese Academy of Sciences, Beijing 100049, China; 3 Beijing Key Lab of CryoBiomedical Engineering, Technical Institute of Physics and Chemistry, Chinese Academy of Sciences, Beijing 100190, China; 4 The Affiliated Qujing Hospital of Kunming Medical University, Qujing, Yunnan Province 655000, China

**Keywords:** Applied sciences, Materials science, Materials synthesis, Nanomaterials

## Abstract

Liquid metals own great potential for making multifunctional advanced materials. Gallium-based alloys may not work well for load-bearing situations because of their high density, susceptibility to oxidation, and leakage at room temperature. By contrast, bismuth-based alloys exhibit self-supporting rigidity and heat-induced softening, making them attractive alternatives. Here, we present a sugar-sacrificial-templating strategy for fabricating lightweight porous bismuth-based liquid alloys. By controlling the sieved particle size of the template, this method enables tailored pore structures while reducing the weight of the original metal by more than half and preserving its electrical conductivity and phase-change thermal response. It also provides electromagnetic shielding and internal space for mass transport. Finite element simulations reveal how the pore network regulates stress concentration and electromagnetic wave scattering. The material remains stable under flexible encapsulation and variable temperatures. This work provides a design strategy for bismuth-based multifunctional materials with load-bearing capacity, mass delivery, and tunable electromagnetic protection.

## Highlights

• Proposes and fabricates porous bismuth-based liquid metal via sugar templating
• Pore size tunes density, conductivity, mechanics, and shielding behavior
• Porous network supports solute transport and electromagnetic shielding
• Elastomers achieved enable reversible heat-triggered rigid-to-flexible switching

## Graphical abstract

**Figure:**
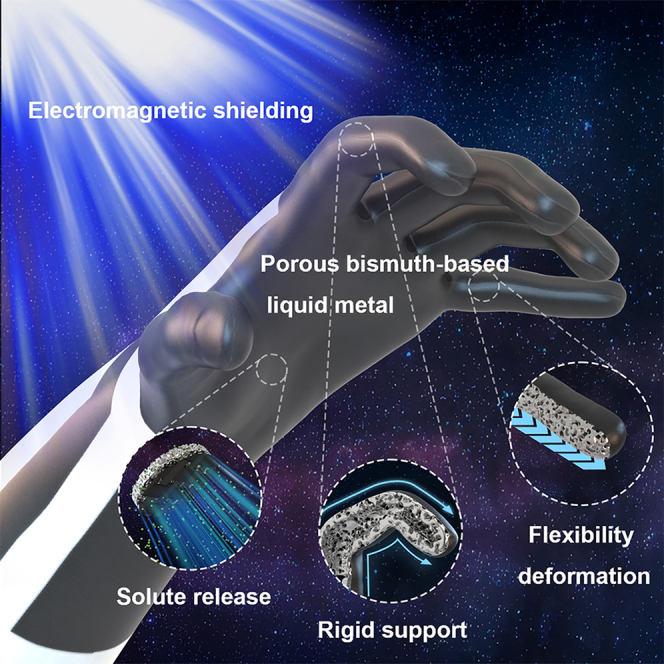

## Introduction

Liquid metals, primarily composed of gallium (Ga), bismuth (Bi), and their alloys, are a class of low-melting-point metallic materials that remain liquid at temperatures below 300 °C.^1^ Liquid metals have attracted increasing attention in flexible electronics,^2^^,^^3^^,^^4^ smart wearables,^5^^,^^6^^,^^7^ and biological engineering^8^^,^^9^^,^^10^^,^^11^^,^^12^^,^^13^^,^^14^ because of their unique solid-liquid phase transition behavior^15^^,^^16^ and excellent physical properties.^17^^,^^18^ Introducing porosity into a continuous metallic phase provides an effective strategy for enhancing the multifunctionality and structural adaptability of liquid metals.^19^ In flexible electronic systems, conventional rigid metals often fail to achieve satisfactory mechanical compatibility with soft biological tissues or deformable substrates.^20^^,^^21^ Incorporating tailored microstructural topologies offers a practical means of alleviating this mismatch.^22^ At the same time, such structural design can substantially reduce the effective density of the material without introducing additional components, thereby enabling lightweight systems and reducing the overall mechanical burden.^23^^,^^24^

Porous phase change metals therefore show considerable potential for flexible electronic applications.^21^^,^^25^^,^^26^ However, current studies have focused primarily on gallium-based alloys that remain liquid at room temperature.^27^^,^^28^^,^^29^^,^^30^^,^^31^^,^^32^^,^^33^^,^^34^ In contrast, the engineering of porous structures in bismuth-based liquid metal systems has been far less investigated, especially regarding the structure-property relationships and multifunctional performance. In this regard, bismuth-based alloys offer several distinct physicochemical advantages,^19^^,^^35^^,^^36^^,^^37^^,^^38^^,^^39^^,^^40^ as summarized in Table S1. Under complex deformation or long-term service, gallium-based materials are prone to challenges such as interfacial oxidation and packaging leakage, which limit their broader use in load-bearing flexible electronic systems. Porous bismuth-based liquid metal (PBLM) can maintain a self-supporting porous framework and good structural stability in the solid state at room temperature.^35^ It also exhibits relatively high stiffness and structural integrity,^41^^,^^42^^,^^43^ while still undergoing phase transition and softening under mild heating.^44^^,^^45^

In addition, the interconnected channels and large specific surface area within the porous network provide sufficient microscopic space for mass transport, energy dissipation, and multiphase integration.^19^ By precisely regulating porosity and pore structure parameters, the mechanical response of the porous material can be tailored to accommodate flexible deformation. This architectural design also enables coordinated optimization of multiple physical properties, including mechanical performance^41^^,^^46^ and thermal transport.^11^^,^^47^^,^^48^ For bismuth-based liquid metals in particular, porous design is also valuable for lightweighting, because their relatively high density compared with gallium-based counterparts can limit their use in weight-sensitive systems (Table S1). In wearable electronic devices, electromagnetic interference has become a critical issue affecting both system stability and user safety. The porous bismuth-based skeleton provides an effective framework for integration with flexible substrates, thereby endowing the overall system with excellent deformability.^26^ Meanwhile, it can also provide electromagnetic shielding capability^49^^,^^50^^,^^51^^,^^52^^,^^53^ and phase-change-enabled variable stiffness,^54^^,^^55^^,^^56^ thereby meeting the coupled mechanical and electrical requirements of shielding components operating under complex deformation conditions.

Here, we present a porous fabrication strategy based on a sugar sacrificial templating method and construct a lightweight porous material from a bismuth-indium-tin alloy. The resulting PBLM markedly reduces effective density and increases specific surface area while retaining the intrinsic advantages of bismuth-based materials, including good electrical conductivity, thermally induced phase transition behavior, and structural stability at room temperature. Microstructural modeling and finite-element simulations further reveal how the designed pore architecture governs the mechanical and electromagnetic responses of the material. In addition, the multifunctional application potential of the porous material is demonstrated in several representative roles, including sustained-release filler media, electromagnetic shielding materials, and deformable composites. Notably, the porous bismuth-based framework endows the composites with both flexibility and thermally tunable rigidity. This work aims to establish and evaluate a lightweight porous Bi-based liquid metal system with distinct structural and functional characteristics, and to provide a useful design reference and initial evaluation basis for the development of bismuth-based liquid metals in multifunctional materials.

## Results

### Preparation

A modified sugar sacrificial templating method was employed in this study to fabricate porous Bi_31.6_In_48.8_Sn_19.6_ alloy material. Compared with conventional foaming or particle filling methods, this strategy does not rely on aqueous electrochemical systems or complex equipment. It is better suited to the characteristics of bismuth-based alloys, which remain solid at room temperature while possessing relatively low melting points that facilitate processing.

**Figure 1:**
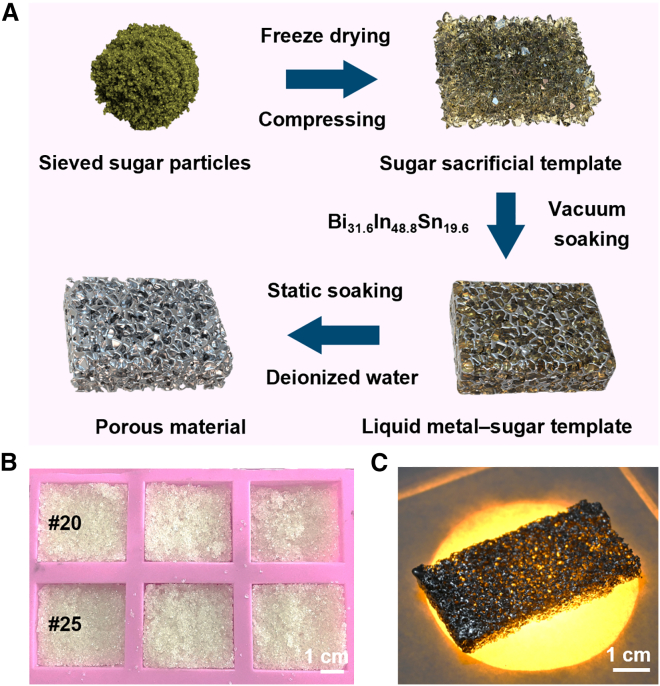
Preparation process and photographs of PBLM fabricated by the sugar sacrificial templating method (A) Schematic illustration of the fabrication procedure for PBLM. (B) Photograph of granulated sugar particles uniformly distributed in the mold. Scale bars, 1 cm. (C) Photograph of PBLM under illumination. Scale bars, 1 cm.

As illustrated in Figure 1A, premium white granulated sugar was first sieved using #20 and #25 standard sieves to obtain template particles with controlled size distributions. The sieved sugar particles were then lightly wetted with a small amount of deionized water at a sugar-to-water mass ratio of 10:1, filled into a silicone mold, compacted, and freeze-dried at −50 °C for 6 h. Through this procedure, sugar sacrificial templates with stable shapes were successfully obtained (Figure 1B). The prepared sugar template was then impregnated with molten liquid metal (Figure S1) for 12 h under continuous vacuum at 0.1 MPa. This process effectively removed trapped gas from the template pores, allowing the liquid metal to fully infiltrate the interparticle gaps under the combined effects of capillary action and pressure difference. Finally, the resulting composite was immersed in deionized water for 24 h to dissolve and remove the sugar phase, thereby releasing the internal porous structure. In this way, a lightweight PBLM with good light transmittance was successfully obtained. Under illumination, the material exhibited a clear and uniformly distributed porous structure (Figure 1C).

### Particle size characterization

**Figure 2:**
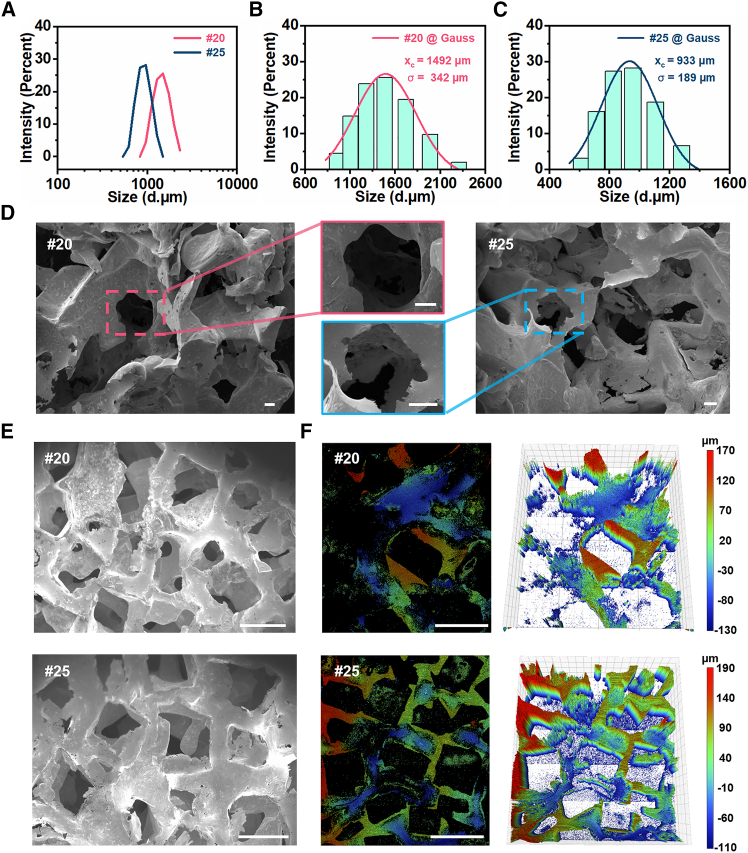
Particle size distributions of the sugar templates and surface morphologies of the resulting PBLMs (A) Particle size comparison of sugar granules retained by the #20 and #25 sieves. (B) Particle size distribution of sugar granules retained by the #20 sieve and the corresponding Gaussian fitting curve. (C) Particle size distribution of sugar granules retained by the #25 sieve and the corresponding Gaussian fitting curve. (D) SEM images of the PBLM samples. Scale bars, 100 μm. (E) Cross-sectional SEM images of the PBLM samples. Scale bars, 1 cm. (F) White-light interferometric 3D morphology of the PBLM samples. Scale bars, 1 cm.

The particle size of the sugar sacrificial template directly determines the microscopic pore structure of PBLM. Sugar particles retained by the #20 and #25 standard sieves were collected and subjected to particle size analysis. The results showed that both groups exhibited unimodal size distributions that closely followed a Gaussian profile (Figure 2A). The particles retained by the #20 sieve exhibited a larger overall size, with a fitted central value of 1.49 mm and a standard deviation of 0.34 mm (Figure 2B), respectively. In comparison, the particles retained by the #25 sieve were distinctly smaller, showing a fitted central value of 0.93 mm and a standard deviation of 0.19 mm (Figure 2C), respectively. Notably, the standard aperture sizes of the #20 and #25 sieves are approximately 0.9 mm and 0.75 mm, respectively, and in both cases are more than one standard deviation smaller than the corresponding fitted central values. This result indicates that the sieving process effectively set the lower size limit of the selected sugar particles, while still preserving a clear size distinction between the two groups. This difference in particle size provided a structural basis for constructing pore units with distinct characteristic dimensions, and the corresponding porous morphologies were shown in Figure S2.

Scanning electron microscopy (SEM) further revealed the relationship between template particle size and the resulting porous morphology (Figure 2D). Both types of samples exhibited a typical porous metallic skeleton. At higher magnification, the inner pore walls displayed irregular polyhedral features, consistent with the interstitial geometry formed by the close packing of sugar particles. In addition, some pores were interconnected through channels generated by local bonding between adjacent sugar particles. A comparison of the two samples revealed clear differences in microstructure. PBLM prepared from the #20 sieve derived template exhibited larger pores, a relatively looser metallic skeleton, and a correspondingly higher pore fraction. In contrast, the sample prepared from the smaller sugar particles retained by the #25 sieve showed a denser pore distribution and a more continuous metal network, indicating higher skeleton integrity and a greater fraction of the liquid metal phase. These morphological differences were consistent with the measured particle size distributions. Cross-sectional SEM images (Figure 2E) further revealed the widespread distribution of pores throughout the internal structure, while white-light interferometry (Figure 2F) provided a complementary three-dimensional view of these porous features.

Overall, the sugar particle sacrificial templating method enables controllable tuning of pore parameters in PBLM. In practical fabrication, the sieve size can be adjusted according to the targeted physical properties, thereby optimizing the characteristic pore size and skeleton morphology of the material.

### Physical property characterization

**Figure 3:**
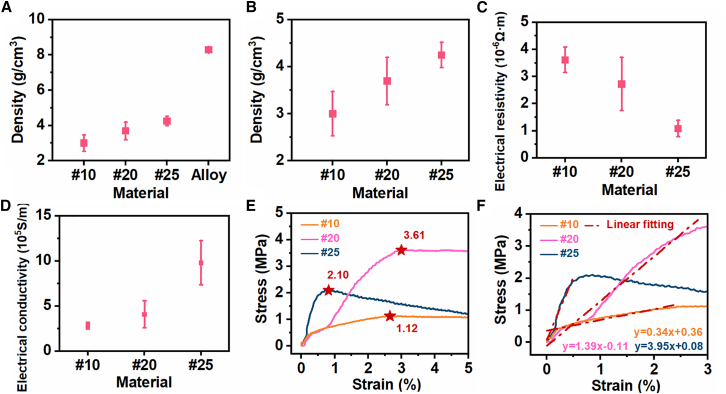
Physical property characterization of PBLM (A–D) Data are represented as mean ± standard deviation. (A) Density comparison between the porous samples and the dense alloy. (B) Density comparison of porous samples prepared with different template particle sizes. (C) Electrical resistivity comparison. (D) Electrical conductivity comparison. (E) Stress-strain curves, with ★ indicating the yield stresses. (F) Fitting results of the stress-strain curves.

To further assess the suitability of PBLM for multifunctional applications, its density, electrical performance, and mechanical properties were systematically evaluated. Attention was given to the influence of the porous architecture on the macroscopic behavior of the material and its potential for practical use. To better define the applicable particle-size range for sacrificial templating, samples prepared using different sugar particle sizes were compared. As shown in Figure 3, template size had a pronounced effect on density, electrical transport, and mechanical response.

Both excessively large and extremely small template particles were unfavorable for the formation of stable PBLM, and this was reflected in the resulting material properties. Accordingly, except for the #20 and #25 groups, two additional conditions were examined: particles retained by the #10 sieve (standard aperture size, 2.0 mm) and particles passing through the #30 sieve (standard aperture size, 0.6 mm).

For the #30 sample, the fine particles tended to aggregate and formed overly narrow interparticle gaps, which hindered infiltration of the high-surface-tension liquid metal. As a result, severe structural defects were observed after removal of the sacrificial template (Figure S3A). This condition was therefore excluded from the subsequent quantitative property comparison. In contrast, the #10 sample could still be prepared as an intact specimen for property testing. It yielded the lowest apparent density (Figures 3A and 3B), but the excessively large pores weakened the metallic framework, leading to increased fragility (Figure S3B) as well as reduced electrical performance (Figures 3C and 3D) and mechanical strength (Figures 3E and 3F). Therefore, the #20 and #25 groups were selected for detailed characterization and further application exploration.

As shown in Figure 3A, the porous structure offered a distinct advantage in reducing weight. The densities of the PBLM samples were substantially lower than that of the dense Bi-In-Sn alloy, with the maximum reduction exceeding one-half. The porosity (*P*) of PBLM was estimated from density measurements according to the following equation^57^:

(Equation 1) $$\begin{array}{c} P=\left(1-\frac{\rho_{\text{PBLM}}}{\rho_{\text{alloy}}}\right)\times 100\% \end{array}$$

where $\rho_{\text{PBLM}}$ and $\rho_{\text{alloy}}$ are the average densities of the porous sample and the corresponding dense alloy, respectively. The average porosity values of the #10, #20, and #25 samples were 63.79 ± 5.73%, 55.43 ± 6.07%, and 48.75 ± 3.24%, respectively. Between the two optimized groups, the sample prepared using the #20 sieve had a lower density than that prepared using the #25 sieve (Figure 3B), which is attributable to the higher porosity generated by the larger template particles.

Despite the introduction of porosity, PBLM retained the high electrical conductivity typical of liquid metals. As shown in Figures 3C and 3D, the optimized samples exhibited electrical conductivities on the order of 10^5^ S/m and resistivities on the order of 10^−6^ Ω m. The #25 sample showed higher conductivity and lower resistivity, which can be explained by its denser framework and more continuous conductive pathways. In addition, the porous structure was introduced without adding any new components, and the DSC results remained stable (Figure S4).

For mechanical characterization, uniaxial tensile tests were performed on PBLM prepared with different template particle sizes. The geometry and dimensions of the tensile specimens are shown in Figure S5, and the corresponding stress-strain curves are presented in Figure 3E. Among the tested groups, the #10 sample exhibited the lowest yield stress (1.12 MPa) and apparent elastic modulus (34 MPa), indicating weakened mechanical support due to the excessively large pores. In contrast, the #20 and #25 samples maintained better mechanical integrity. Both optimized samples displayed typical deformation behavior, consisting of an initial elastic region followed by a nonlinear response. The yield stresses of the #20 and #25 samples were 3.61 and 2.10 MPa, respectively, while the corresponding apparent elastic moduli were 139 and 395 MPa (Figure 3F). The #25 sample exhibited higher stiffness but slightly lower strength, which may be related to the more pronounced stress concentration associated with the finer pore structure.

### Theoretical model and simulation

**Figure 4:**
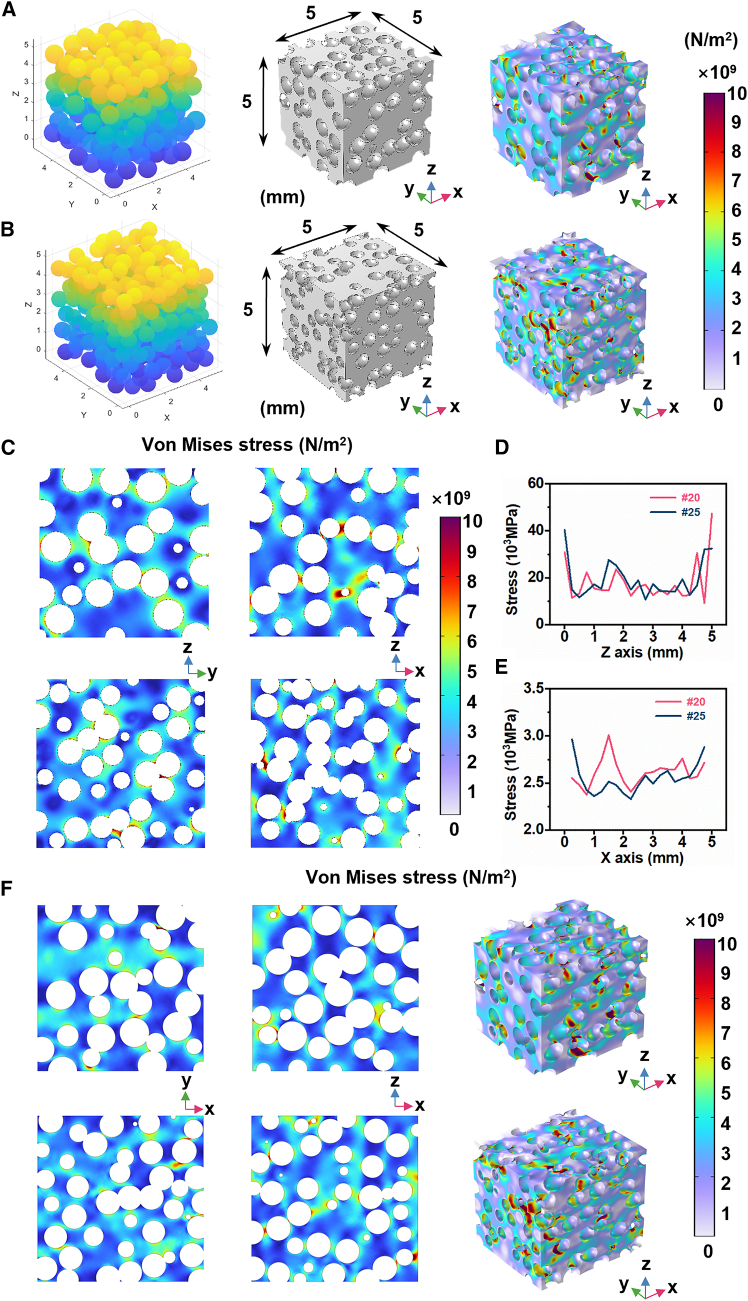
Finite element simulation and stress analysis of PBLM (A) Structural model and overall stress distribution under compression for the porous sample generated from the #20 sieve particle size distribution. (B) Structural model and overall stress distribution under compression for the porous sample generated from the #25 sieve particle size distribution. (C) von Mises stress distribution under compression along the longitudinal section at *x* = 2.5 mm or *y* = 2.5 mm for models with different particle-size distributions (upper for #20, lower for #25). (D) Comparison of the maximum von Mises stress across cross sections along the *z* direction under compression. (E) Comparison of the average von Mises stress across cross sections along the *x* direction under compression. (F) von Mises stress distribution under tension along the transverse section at *z* = 2 mm and the longitudinal section at *y* = 2 mm for models with different particle-size distributions (upper for #20, lower for #25).

To better understand the microstructure-dependent mechanical behavior of PBLM, structural models were constructed for finite element simulation. The models were generated by random sphere packing combined with k-dimensional data structure storage, with the spheres representing the sugar particles in the sacrificial template. A maximum relative tolerance of 10% was introduced into the collision detection criterion to mimic the moisture-induced adhesion between adjacent sugar particles. The porous network model was then generated by Boolean subtraction of the sphere domain from the liquid metal domain. Figures 4A and 4B showed the sphere distributions derived from the sugar templates prepared using the #20 and #25 sieves, the corresponding porous models used for simulation, and the results of the uniaxial compression analysis. The equivalent von Mises stress^58^ $\sigma_{\text{vm}}$ was calculated using the principal stresses $\sigma_{1}$, $\sigma_{2}$, $\sigma_{3}$:

(Equation 2) $$\begin{array}{c} \sigma_{\text{vm}}=\sqrt{\frac{1}{2}\left[{\left(\sigma_{1}-\sigma_{2}\right)}^{2}+{\left(\sigma_{2}-\sigma_{3}\right)}^{2}+{\left(\sigma_{3}-\sigma_{1}\right)}^{2}\right]} \end{array}$$

As a result, both models exhibited distinctly non-uniform stress distributions under compression, with stress mainly concentrated in the thin wall regions surrounding the pores. As shown in Figure 4C, the longitudinal section along the central axis provides a clearer view of how the pore structure influences stress transfer within the bismuth-based liquid metal framework. In the #20 model, the pores were larger and the void volume fraction was higher, resulting in a smaller effective load-bearing cross section, lower overall stiffness, and a more dispersed pattern of stress concentration. In contrast, the #25 model contained more pores with smaller spacing, which tended to form narrower and more continuous metallic skeletons. Consequently, stress concentration developed more readily during compression, leading to a greater number of high-stress regions.

To further characterize the internal stress distribution under compression, a series of equally spaced cross sections along the vertical z and x directions was analyzed, and the maximum and average von Mises stresses of each section were extracted. The numerical results further showed that, across the full set of z-direction sections, the #20 model exhibited fewer stress peaks than the #25 model, as indicated by the smaller number of sharp peaks in Figure 4D. Meanwhile, the average stress of the #20 model was generally higher across the x-direction sections, as shown in Figure 4E.

In addition, uniaxial tensile simulation was performed for the same porous models, and the results are presented in Figure 4F. The stress distribution is shown along both the transverse and longitudinal sections. Similar trends were observed under tension: the #20 model, with a lower fraction of load-bearing metallic phase, exhibited a broader and more dispersed region of elevated stress, whereas the #25 model showed a larger number of more localized high-stress regions.

It should be noted that the simulation model is an idealized representation of the real samples, particularly with respect to sphere arrangement and geometry conditions. Therefore, these simulation results are better suited for qualitatively illustrating stress evolution within the porous structure under mechanical loading, rather than for direct quantitative comparison with the experimental results.

### Solute uptake and release behavior

**Figure 5:**
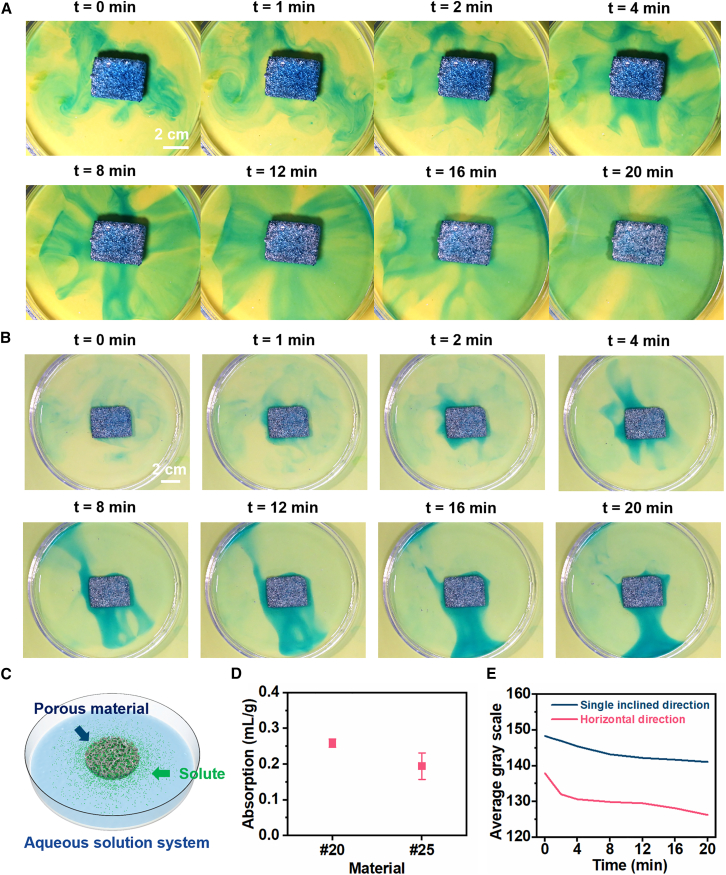
Solute uptake and release behavior of PBLM (A) Time evolution of colored liquid release in the horizontal direction. Scale bars, 2 cm. (B) Time evolution of colored liquid release in the inclined direction. Scale bars, 2 cm. (C) Schematic illustration of the solute release process. (D) Characterization of the solute uptake performance. Data are represented as mean ± standard deviation. (E) Time-dependent variation in average grayscale during the release process.

As a loadable porous structure, the uptake and release behavior of PBLM provided direct evidence of its interconnected internal pore network and indicated its basic capability for solute transport. This behavior was demonstrated through a laboratory-scale demonstration. As shown in Figures 5A and 5B, dyed ink continuously diffused outward from the interior of the porous sample (#20), forming distinct color gradients and diffusion plumes. The spatial spreading of the ink throughout the surrounding region could also be observed in Figure S6. These observations directly confirmed the continuous interconnection of the internal pore channels, which provided effective pathways for solute infiltration, retention, and outward transport. The schematic illustration in Figure 5C further clarifies this process.

The quantitative results were consistent with these observations. Figure 5D shows that both samples prepared with different template particle sizes exhibited solute uptake capability, while the #20 sample displayed a higher uptake, further indicating the storage and transport function enabled by the interconnected pore channels. In addition, Figure 5E quantified the release process through grayscale analysis, showing that the colored component was released continuously and steadily over time. This result further confirmed the connectivity of the pore network and its effectiveness in mass transfer.

Taken together, these results indicated that the interconnected porous structure of PBLM can support solute uptake and release at a qualitative level, while demonstrating pore interconnectivity and basic transport capability. Such structural features may provide a useful basis for future exploration in functional loading and related transport-based material designs.

### Electromagnetic shielding performance

**Figure 6:**
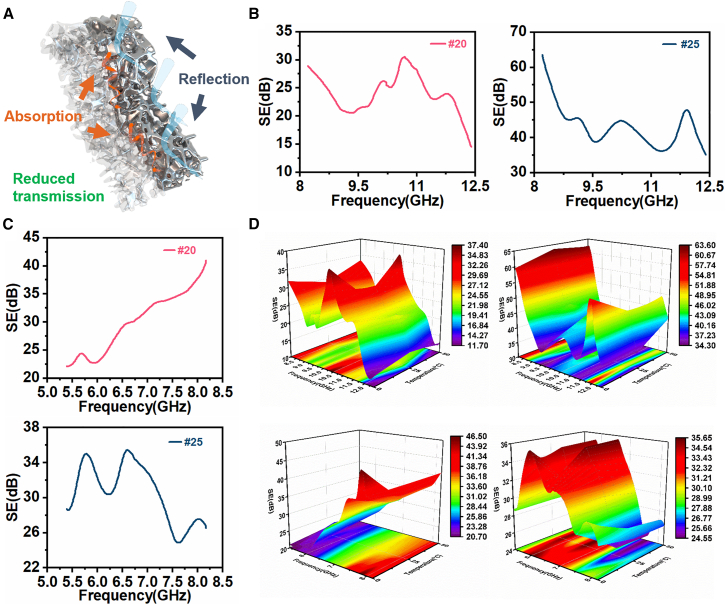
Electromagnetic shielding performance of PBLM (A) Schematic illustration of the shielding mechanism. (B) Shielding effectiveness in the X band. (C) Shielding effectiveness in the 5–8 GHz range within the C band. (D) Shielding effectiveness under different thermal histories.

As a structurally modified metallic functional material, PBLM also showed considerable promise for electromagnetic shielding owing to the introduction of porosity. As illustrated in Figure 6A, its shielding mechanism can be understood as a synergistic process dominated by reflection and supplemented by absorption. The continuous metallic phase provided relatively high electrical conductivity, leading to strong reflection of incident electromagnetic waves because of interfacial impedance mismatch.

Meanwhile, the porous architecture introduced abundant internal interfaces, promoted multiple scattering, and prolonged the propagation paths of electromagnetic waves within PBLM.^59^^,^^60^^,^^61^^,^^62^^,^^63^^,^^64^ In addition, the porous network could improve local impedance matching, allowing more incident waves to penetrate the interior rather than being directly reflected at the surface.^59^^,^^65^^,^^66^^,^^67^^,^^68^^,^^69^ The heterogeneous solid-air interfaces and structural irregularities associated with the porous framework could also induce interfacial and dipolar polarization, while the continuous conductive framework may further contribute to conduction-related energy dissipation,^60^^,^^61^^,^^62^^,^^65^ thereby promoting electromagnetic energy dissipation and enhancing the absorption contribution.^70^ Similar mechanisms have also been reported for other porous electromagnetic materials.^59^^,^^60^^,^^61^^,^^62^^,^^63^^,^^64^^,^^65^^,^^66^^,^^67^^,^^68^^,^^69^^,^^70^

For the X band (8–12 GHz), the electromagnetic shielding effectiveness (SE) of PBLM samples with a thickness of 5 mm was measured at room temperature. A schematic illustration of the measurement setup is provided in Figure S7A. During testing, the sample was mounted in a waveguide-based fixture connected to a vector network analyzer, with the transmitting and receiving ports located on opposite sides. Before measurement, the vector network analyzer was calibrated using direct-connection, short-circuit, and quarter-waveguide standards, and the reference plane was set at the two fixture interfaces. The sample geometry was matched to the holder aperture to fully cover the rectangular test window inside the fixture. The PBLM samples in this section were tested without encapsulation to preserve their porous structure, and the sample perimeter was tightly pressed against the fixture walls by the holder during measurement to ensure a tight seal and close contact. In the following section, the same setup was used to evaluate the encapsulated PBLM-elastomer composites. The SE was calculated according to the following equation^71^:

(Equation 3) $$\begin{array}{c} SE\left(\text{dB}\right)=-10\cdot \log_{10}{\left|S_{21}\right|}^{2} \end{array}$$

where |*S*_21_| is the transmission coefficient measured by the vector network analyzer. As shown in Figure 6B, both samples exhibited stable shielding performance over the tested frequency range, with the overall transmittance remaining below 0.5%. The #25 sample showed superior shielding performance, which could be attributed to its higher volume fraction of the metallic phase. Measurements in the 5–8 GHz range, which lies within the standard C band (4–8 GHz), further confirmed the good frequency adaptability of the material (Figure 6C). In addition, thermal history tests, including cooling to 0 °C and heating to 50 °C with a holding time of 1 h, showed only small variations in SE and good overall stability below the melting point (Figure 6D). By contrast, shielding stability during melt-solidify cycling across the phase transition was further evaluated in the elastomer-encapsulated configuration, as described in the following section. These results support the potential of the material for multifunctional applications such as wearable electronic systems, where lightweight design, environmental stability, and integrated shielding functionality are simultaneously required.^68^^,^^70^

To further clarify the effect of the porous structure, the electromagnetic SE of dense Bi_31.6_In_48.8_Sn_19.6_ alloy with a thickness of 5 mm was additionally measured, and the result is shown in Figure S7B. By comparison, although the introduction of porosity led to a reduction in SE, PBLM still retained a meaningful level of electromagnetic shielding capability while offering the distinct advantage of reduced weight through its interconnected porous architecture.

**Figure 7:**
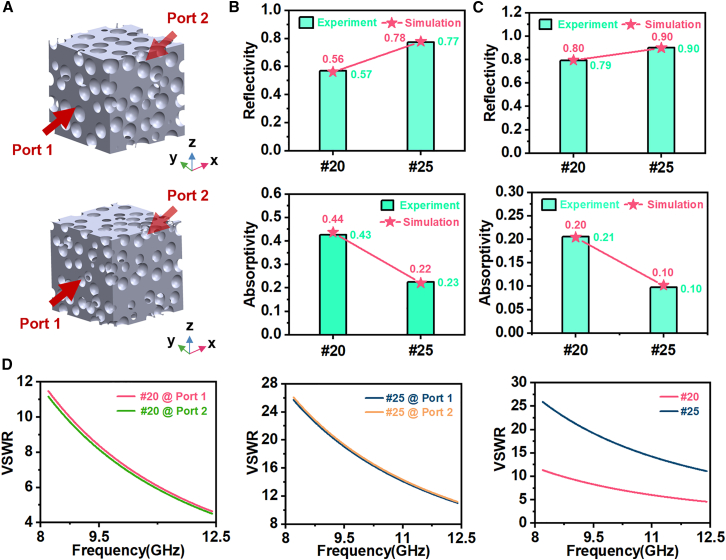
Electromagnetic simulation model and shielding characteristics of PBLM (A) CST simulation model (upper for #20, below for #25). (B) Simulated and experimental reflectivity and absorptivity in the X band. (C) Simulated and experimental reflectivity and absorptivity in the 5–8 GHz range within the C band. (D) VSWR of models with different template particle sizes.

To clarify the role of porosity in the electromagnetic shielding behavior of PBLM from the perspective of electromagnetic wave propagation, three-dimensional porous models with geometric features representative of the actual samples were constructed in Computer Simulation Technology (CST) Studio Suite, and the scattering parameters under port excitation were calculated. As idealized geometric representations, these models were used here to provide qualitative insight into the electromagnetic shielding behavior of PBLM. The pore structures of the models were parameterized according to the template particle sizes corresponding to the #20 and #25 samples. The geometric configurations of the models and the incident port setup are shown in Figure 7A. In the CST simulations, the electrical conductivity of the dense Bi-In-Sn alloy was determined from the measured resistivity, and the relative permittivity was derived accordingly. Since the alloy is non-magnetic, its relative magnetic permeability was set to 1. Waveguide ports were assigned to both excitation ends, and bidirectional excitation along the positive and negative x directions was employed to evaluate the influence of incident direction. Electric boundary conditions were applied to the two faces normal to the port direction, while the remaining boundaries were defined as open boundaries. The solver convergence criterion was set to 10^−4^.

Based on these models, the shielding performance in the X band and the 5–8 GHz range within the C band was calculated and systematically compared with the experimental results obtained over the same frequency range. The reflection coefficient *R*, transmission coefficient *T*, and absorption coefficient *A* were calculated according to the following equation^72^:

(Equation 4) $$\begin{array}{c} R={\left|S_{11}\right|}^{2} \end{array}$$

(Equation 5) $$\begin{array}{c} T={\left|S_{21}\right|}^{2} \end{array}$$

(Equation 6) $$\begin{array}{c} A=1-R-T \end{array}$$

where *R*, *T* and *A* represent the reflection, transmission, and absorption coefficients, respectively, while $\left|S_{11}\right|$ and $\left|S_{21}\right|$ denote the magnitudes of the reflection and transmission scattering parameters. The reflectivity and absorptivity obtained from both experiment and simulation are presented in Figures 7B and 7C. The results showed good agreement between simulation and experiment. Both models exhibited reflection-dominated shielding behavior, while the #20 model showed a relatively greater absorption contribution. This difference was closely related to the pore-size-dependent continuity of the metallic skeleton. To further quantify the reflection behavior and impedance matching, the voltage standing wave ratio (VSWR) was calculated according to the following equation^72^:

(Equation 7) $$\begin{array}{c} \text{VSWR}=\frac{1+\left|S_{11}\right|}{1-\left|S_{11}\right|} \end{array}$$

where |*S*_11_| is the magnitude of the reflection coefficient at the input port. As shown in Figure 7D, the bidirectional responses of each model with the same pore size were generally consistent, while the #25 model exhibited a higher VSWR and stronger reflection.

Overall, the model effectively captured the influence of pore size on the shielding mechanism, confirmed the reliability of the simulation, and provided a useful basis for parameter selection in engineering design.

### Flexible PBLM-elastomer composite

A composite system based on PBLM and elastomer was further constructed, exhibiting distinctive mechanical advantages through the synergistic interaction of the two components. Owing to its high specific surface area and interconnected porous structure, PBLM provided abundant and stable interfacial sites for bonding with the elastomer, thereby helping maintain the integrity of the composite architecture.

**Figure 8:**
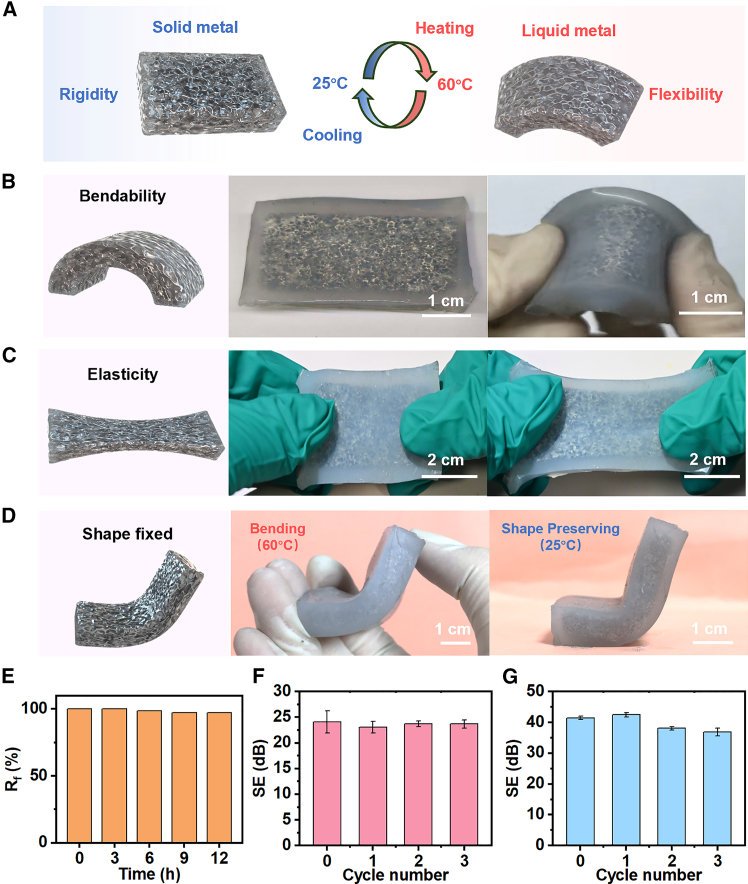
Flexible and thermally responsive mechanical behavior of the PBLM-elastomer composite (A–E) show the results for the #25 samples. (A) Reversible switching between rigid and flexible states under temperature variation. (B) Bending behavior at elevated temperature. Scale bars, 1 cm. (C) Tensile deformation and reversibility at 60 °C. Scale bars, 2 cm. (D) Shape fixing behavior at elevated temperature followed by shape preservation at room temperature. Scale bars, 1 cm. (E) Shape fixity ratio at room temperature. (F) Shielding effectiveness of the #20 samples with repeated melting-solidification cycles. Data are represented as mean ± standard deviation. (G) Shielding effectiveness of the #25 samples with repeated melting-solidification cycles. Data are represented as mean ± standard deviation.

The temperature-controlled experiment was conducted to evaluate the feasibility of this composite system for practical use. At room temperature (25 °C), the bismuth-based liquid metal remained in the solid state and acted as a stable reinforcing framework for the elastomer, giving the composite an overall rigid character. When the temperature was increased to 60 °C, the metal underwent a phase transition to the liquid state, and the composite correspondingly became highly flexible through its interaction with the elastomer. This reversible transition between rigid and flexible mechanical states could be repeatedly achieved by heating and cooling, and the corresponding behavior and mechanism are illustrated in Figure 8A.

Figure 8B directly showed the flexible state of the PBLM-elastomer composite at elevated temperature, where it can be freely bent into an arc while maintaining structural integrity. During bending, aided by elastomer encapsulation and the redistribution of liquid metal within the composite, no fracture of the elastomer or leakage of the bismuth-based liquid metal was observed, indicating good conformability to curved surfaces and structural stability. Figure 8C further demonstrated the tensile elasticity of the composite at elevated temperature, as it recovered its original shape after removal of the external tensile force, reflecting good deformation reversibility. The corresponding dynamic flexible behavior is provided in Video S1. In addition, the composite exhibited typical shape-fixing behavior.^73^ As shown in Figure 8D, the sample was deformed into a temporary shape at 60 °C and then cooled to 25 °C. After solidification, the external support was removed, while the deformed shape remained well retained, demonstrating stable temporary shape fixation. The shape stability was quantified using the shape fixity ratio^73^:

(Equation 8) $$\begin{array}{c} R_{f}=\frac{\theta_{f\left(t\right)}}{\theta_{i}}\times 100\% \end{array}$$

where *θᵢ* is the bending angle of the initially fixed temporary shape, and *θ*_*f(t)*_ is the corresponding angle measured after holding at room temperature for a given period. As shown in Figure 8E, the composite exhibited stable retention over time, further confirming its ability to preserve the temporary shape after thermal triggering.

Video S1. Tensile and bending behavior of porous bismuth-based liquid metal material, related to Figure 8

In terms of electromagnetic shielding performance, repeated melting-solidification cycling tests were performed on the PBLM-elastomer composites with a PBLM layer thickness of 5 mm. In each cycle, the samples were heated to 80 °C and held for 30 min to ensure complete melting, followed by storage at room temperature for 60 min to allow full solidification. The SE was then evaluated during multiple cycles, as shown in Figures 8F and 8G. The samples maintained stable shielding performance during repeated phase-transition cycling, indicating good thermal stability of the PBLM-elastomer composites.

Overall, the thermally reversible switching between rigid and flexible states, together with the favorable mechanical performance and shape-fixing behavior, provides important support for the structural design and dynamic adaptation of flexible electromagnetic shielding materials under varying temperature conditions. These characteristics also broaden the application potential of liquid metal materials in electromagnetic protection. The porous structure of PBLM greatly lowers the density of the Bi-based alloy, enabling it to maintain good electromagnetic shielding performance under weight-sensitive conditions. The absolute SE (SSE/t) was calculated according to the following equation^74^:

(Equation 9) $$\begin{array}{c} \text{SSE}/t=\frac{\text{SE}}{\rho \times t} \end{array}$$

where ρ is the density of the sample and *t* is the sample thickness. As shown in Figure S8, the introduction of the porous structure markedly improves the absolute SE of the original Bi-In-Sn alloy, allowing PBLM to achieve a competitive level comparable to that of many commonly reported shielding materials.^75^^,^^76^^,^^77^^,^^78^

## Discussion

To address the load-bearing limitations and leakage risks of traditional gallium alloys, a PBLM was proposed and fabricated using the sugar templating method, with the pore architecture regulated by different template sizes. PBLM exhibited substantially lower density while retaining high electrical conductivity, tunable mechanical properties, solute release capability, and stable electromagnetic shielding performance. Experimental characterization revealed clear structure-property relationships: The sample with larger pores showed higher liquid uptake and a greater absorption contribution to electromagnetic shielding, whereas the sample with finer pores exhibited higher conductivity, greater stiffness, and stronger reflection capability. To clarify the underlying mechanisms, structural modeling was carried out using MATLAB, combined with simulations performed in COMSOL and CST Studio Suite. The simulation results provided qualitative support for the observed trends and effectively explained the stress concentration behavior and shielding mechanism involving reflection and absorption. In addition, a PBLM-elastomer composite was developed, showing temperature-responsive switching between rigid and flexible states. Overall, this study provides an effective structural design strategy for lightweight bismuth-based liquid metals and offers useful guidance for the development of multifunctional, tunable materials for flexible electronics and wearable shielding.

### Limitations of the study

This work serves as an initial exploration of the construction strategy and application potential of the porous bismuth-based liquid metal material for structural electronic applications. However, some limitations remain to be addressed in future research. The influence of alloy composition on the properties of bismuth-based liquid metals was not systematically investigated, particularly with respect to variations in liquid metal ratio and the resulting changes in melting behavior. In addition, the electromagnetic shielding performance of the porous material was evaluated only in the X band and C band. Further studies over a broader frequency range are needed to more comprehensively assess its shielding capability and application potential.

## Resource availability

### Lead contact

Further information and requests for resources and reagents should be directed to and will be fulfilled by the lead contact, Jing Liu (jliu@mail.ipc.ac.cn).

### Materials availability

This study did not generate new unique reagents.

### Data and code availability

• Data reported in this article will be shared by the lead contact on request.
• All original code is available in this study’s supplemental information.
• Any additional information required to reanalyze the data reported in this study is available from the lead contact upon request.

## Acknowledgments

This work was partially supported by the Yunnan Science and Technology Talent and Platform Program (Academician and Expert Workstation), Liu Jing Expert Workstation (202205AF150054).

## Author contributions

J.W., Y.W., and Y.B.: conceptualization, methodology, investigation, data curation, formal analysis, validation, visualization, writing – original draft, and writing – review and editing; J.L., J.Z., and M.G.: investigation; Z.D., Y.Z., and W.R: methodology and supervision; J.Z.: writing – original draft; J.L.: conceptualization, visualization, supervision, writing – review and editing, and funding acquisition. All authors read and approved this manuscript.

## Declaration of interests

The authors have no conflicts to disclose.

## STAR★Methods

### Key resources table

**Table:** 

REAGENT or RESOURCE	SOURCE	IDENTIFIER
**Chemicals, peptides, and recombinant proteins**		

Bismuth (Bi)	Zhuzhou Yilong New Materials Co., Ltd.	2311774
Indium (In)	Zhuzhou Smelter Group Co., Ltd.	2311800
Tin (Sn)	Zhuzhou Yilong New Materials Co., Ltd.	2311418
Ecoflex 00-30	Smooth-On	FYS0030

**Software and algorithms**		

MATLAB R2024b	MathWorks	www.mathworks.com
OriginPro 2024	OriginLab	www.originlab.com
COMSOL Multiphysics 6.3	COMSOL	www.comsol.com
CST Studio Suite 2024	Dassault Systèmes	www.3ds.com

### Experimental model and study participant details

This study did not involve experimental models or study participants, including animals, human participants, plants, microbe strains, cell lines, or primary cell cultures. No human-derived samples were used. Therefore, sex and gender were not considered.

### Method details

#### Materials

Bismuth (Bi) and tin (Sn) with a purity of 99.99% were supplied by Zhuzhou Yilong New Materials Co., Ltd. (Zhuzhou, China). Indium (In) with a purity of 99.99% was supplied by Zhuzhou Smelter Group Co., Ltd. (Zhuzhou, China). Ecoflex 00-30 was supplied by Smooth-On (USA), used for preparing the elastomer. Premium white granulated sugar was supplied by Aladdin Biochemical Technology Co., Ltd. (Shanghai, China), used for preparing the sugar template. Blue compound colorant was supplied by Guangzhou Fuzheng Donghai Food Co., Ltd. (Guangzhou, China), used for solution staining.

#### Characterization and measurements

(1) Mechanical vibrating sieve shaker, used for sieving granulated sugar to obtain sugar particles with different particle-size ranges. Instrument No.: 890218100138, Shanghai Yidian Physical Optical Instrument Co., Ltd. Rated power: 150 W. (2) Laboratory basic freeze dryer, used for freeze-drying and shaping the formed sugar molds to prepare the sacrificial sugar templates for subsequent liquid metal infiltration. Model: L3-65, Changsha Kaipu Instrument Co., Ltd. (3) Vacuum drying oven, used for promoting the full infiltration, penetration, and filling of the pores in the sugar template by the alloy under heating and vacuum conditions, thereby preparing the liquid metal-sugar template composite. Model: DZF, Beijing Yongguangming Medical Instrument Co., Ltd. Maximum vacuum degree: 0.1 MPa. (4) Electronic balance, used for weighing the metal raw materials. Model: ZN-C50002, Hangzhou Youheng Weighing Equipment Co., Ltd. Resolution: 0.01 g. Maximum capacity: 5000 g. (5) Differential scanning calorimeter, used for measuring the phase-transition temperature and phase-change enthalpy of the porous bismuth-based liquid metal. Model: NETZSCH DSC 200 F3 Maia, NETZSCH, Germany. (6) Intelligent heating platform, used for heating the metal alloy. Model: GJR-2020, Hangzhou Gongjiangren Technology Co., Ltd. (7) Tensile testing platform, used for uniaxial tensile tests of the lightweight porous bismuth-based liquid metal samples. Uniaxial tensile tests were carried out at room temperature at a crosshead speed of 100 mm min^-1^ until fracture. Strain was calculated from the crosshead displacement, and tensile force was measured using a load-cell sensor. The Young’s modulus and tensile strength were determined from the resulting stress-strain curves. Model: TJL-1, Anhui Tianguang Sensor Technology Co., Ltd. (8) Nanovoltmeter, used for measuring the electrical resistance and conductivity of the samples. Model: 34420A, Agilent Technologies. (9) Laser particle size analyzer, used for measuring the particle-size distribution of the sugar particles. Model: Zetasizer Nano ZSE. (10) Vector network analyzer, used for real-time recording of the electromagnetic shielding effectiveness of the porous liquid metal samples during testing. Model: MS4642, Anritsu. (11) White-light interferometric 3D profilometer, used to characterize the porous structural features. Model: Contour Elite I.

### Quantification and statistical analysis

No data pre-processing was performed. No statistical significance tests were performed. Data in Figures 3A–3D, 5D, 8F, and 8G were presented as mean +/- standard deviation (SD). For other figures, raw data were presented without transformation. Statistical methods included Gaussian distribution fitting for Figures 2B and 2C, and linear regression fitting for the data in Figure 3F. All statistical analyses were conducted using the built-in data fitting tools in Origin software.

## References

[1] Daeneke T., Khoshmanesh K., Mahmood N., de Castro I.A., Esrafilzadeh D., Barrow S.J., Dickey M.D., Kalantar-Zadeh K. (2018). Liquid metals: fundamentals and applications in chemistry. Chem. Soc. Rev..

[2] Chen S., Wang H., Zhao R., Rao W., Liu J. (2020). Liquid metal composites. Matter.

[3] Haque A., Tutika R., Byrum R.L., Bartlett M.D. (2020). Liquid metal microstructures: Programmable liquid metal microstructures for multifunctional soft thermal composites. Adv. Funct. Mater..

[4] Guymon G.G., Malakooti M.H. (2022). Multifunctional liquid metal polymer composites. J. Polym. Sci..

[5] Carey B.J., Ou J.Z., Clark R.M., Berean K.J., Zavabeti A., Chesman A.S., Russo S.P., Lau D.W., Xu Z., Bao Q. (2017). Wafer-scale two-dimensional semiconductors from printed oxide skin of liquid metals. Nat. Commun..

[6] Li Q., Lin J., Liu T., Zhu X., Yao W., Liu J. (2021). Gas-mediated liquid metal printing toward large-scale 2D semiconductors and ultraviolet photodetector. npj 2D Mater. Appl..

[7] Nguyen C.K., Low M.X., Zavabeti A., Jannat A., Murdoch B.J., Della Gaspera E., Orrell-Trigg R., Walia S., Elbourne A., Truong V.K. (2021). Ultrathin oxysulfide semiconductors from liquid metal: a wet chemical approach. J. Mater. Chem. C.

[8] Wang X., Guo R., Liu J. (2019). Liquid metal based soft robotics: materials, designs, and applications. Adv. Mater. Technol..

[9] Wang D., Ye J., Bai Y., Yang F., Zhang J., Rao W., Liu J. (2023). Liquid metal combinatorics toward materials discovery. Adv. Mater..

[10] Yi L., Jin C., Wang L., Liu J. (2014). Liquid-solid phase transition alloy as reversible and rapid molding bone cement. Biomaterials.

[11] Zhang M., Wang X., Huang Z., Rao W. (2020). Liquid metal based flexible and implantable biosensors. Biosensors.

[12] Duan M., Zhu X., Fan L., He Y., Yang C., Guo R., Chen S., Sun X., Liu J. (2022). Phase-transitional bismuth-based metals enable rapid embolotherapy, hyperthermia, and biomedical imaging. Adv. Mater..

[13] Saad M., ALMohiy H., Alqahtani M.S., Alshihri A.A., Shalaby R.M. (2022). Study of structural, physical, characteristics and radiation shielding parameters of Bi50-Pb40-Sn10 and Bi40-Pb40-Sn10-Cd10 alloys used for radiation therapy. Radiat. Eff. Defect Solid.

[14] Liu S., Li L., Jiang C., Wang Q., Deng Z. (2024). Synergistic injection of the thermosensitive hydrogel and Bi-based alloy bone cement for orthopaedic repair. Sci. China Technol. Sci..

[15] Yuan B., Zhao C., Sun X., Liu J. (2019). Liquid-metal-enhanced wire mesh as a stiffness variable material for making soft robotics. Adv. Eng. Mater..

[16] Zhang M., Chen X., Sun Y., Gan M., Liu M., Tang S., Zhang S., Li X., Li W., Sun L. (2023). A magnetically and thermally controlled liquid metal variable stiffness material. Adv. Eng. Mater..

[17] Deng Y., Jiang Y., Liu J. (2021). Low-melting-point liquid metal convective heat transfer: A review. Appl. Therm. Eng..

[18] Ding Y., Liu J. (2016). Water film coated composite liquid metal marble and its fluidic impact dynamics phenomenon. Front. Energy.

[19] Gao J., Ye J., Chen S., Gong J., Wang Q., Liu J. (2021). Liquid metal foaming via decomposition agents. ACS Appl. Mater. Interfaces.

[20] Wang L., Lai R., Zhang L., Zeng M., Fu L. (2022). Emerging liquid metal biomaterials: From design to application. Adv. Mater..

[21] Wang X., Fan L., Zhang J., Sun X., Chang H., Yuan B., Guo R., Duan M., Liu J. (2019). Printed conformable liquid metal e-skin-enabled spatiotemporally controlled bioelectromagnetics for wireless multisite tumor therapy. Adv. Funct. Mater..

[22] Yu D., Liao Y., Song Y., Wang S., Wan H., Zeng Y., Yin T., Yang W., He Z. (2020). A super-stretchable liquid metal foamed elastomer for tunable control of electromagnetic waves and thermal transport. Adv. Sci..

[23] Jaggers R.W., Chen R., Bon S.A. (2016). Control of vesicle membrane permeability with catalytic particles. Mater. Horiz..

[24] Yuan B., Zhao C., Sun X., Liu J. (2020). Lightweight liquid metal entity. Adv. Funct. Mater..

[25] Zhang H., Li C., Li Y., Sun X., Li Q., Gui L. (2024). Micro-casting/assembly technology based on bismuth-based liquid metal. Adv. Mater. Technol..

[26] Zhang M., Zhang P., Zhang C., Wang Y., Chang H., Rao W. (2020). Porous and anisotropic liquid metal composites with tunable reflection ratio for low-temperature electromagnetic interference shielding. Appl. Mater. Today.

[27] Tutika R., Zhou S.H., Napolitano R.E., Bartlett M.D. (2018). Mechanical and functional tradeoffs in multiphase liquid metal, solid particle soft composites. Adv. Funct. Mater..

[28] Krings E.J., Zhang H., Sarin S., Shield J.E., Ryu S., Markvicka E.J. (2021). Lightweight, thermally conductive liquid metal elastomer composite with independently controllable thermal conductivity and density. Small.

[29] Liu Y., Ji X., Liang J. (2021). Rupture stress of liquid metal nanoparticles and their applications in stretchable conductors and dielectrics. npj Flexible Electron..

[30] Yang P., Li X., Yang X., Li G., Hu Z., Huang L., Wu Y. (2022). Lightweight liquid metal-elastomer foam with smart multi-function. Adv. Funct. Mater..

[31] Wang J., Hao Y., Yao Y., Li J., Song Y., Gao J., Liu J. (2024). Stretchable stiffness-tuning of liquid metal elastomer triggered by homocrystal seeds. Appl. Phys. Rev..

[32] Ma J., Liu Z., Nguyen Q.K., Zhang P. (2024). Lightweight soft conductive composites embedded with liquid metal fiber networks. Adv. Funct. Mater..

[33] Ma K., Liu J. (2007). Nano liquid-metal fluid as ultimate coolant. Phys. Lett..

[34] Park Y., Min H., Kim H., Zhexembekova A., Lee C.Y., Park J.-U. (2019). Three-dimensional, high-resolution printing of carbon nanotube/liquid metal composites with mechanical and electrical reinforcement. Nano Lett..

[35] Zhang X., Liu J., Deng Z. (2024). Bismuth-based liquid metals: advances, applications, and prospects. Mater. Horiz..

[36] Song J., Lui T., Chang Y., Chen L. (2005). Compositional effects on the microstructure and vibration fracture properties of Sn–Zn–Bi alloys. J. Alloys Compd..

[37] Gao J., Chen S., Liu T., Ye J., Liu J. (2021). Additive manufacture of low melting point metal porous materials: Capabilities, potential applications and challenges. Mater. Today.

[38] Zhang Q., Yao Y., Gao J., Yang X., Zhang P., Deng Z., Liu J. (2020). Thermal evaluation of the injectable liquid metal bone cement in orthopedic treatment. Sci. China Technol. Sci..

[39] Shan W., Lu T., Majidi C. (2013). Soft-matter composites with electrically tunable elastic rigidity. Smart Mater. Struct..

[40] Liu J., Sheng L., He Z. (2018).

[41] Kamal M., Mazen S., El-Bediwi A.B., Kashita E. (2005). Characterization of bismuth–tin–lead and bismuth–tin–lead–cadmium fusible alloys. Radiat. Eff. Defect Solid.

[42] Hirata Y., Yang C.-h., Lin S.-k., Nishikawa H. (2021). Improvements in mechanical properties of Sn–Bi alloys with addition of Zn and In. Materials Science and Engineering: A.

[43] Hou Y., Myung D., Park J.K., Min J., Lee H.-R., El-Aty A.A., Lee M.-G. (2023). A review of characterization and modelling approaches for sheet metal forming of lightweight metallic materials. Materials.

[44] Pauliukaitė R., Hočevar S.B., Ogorevc B., Wang J. (2004). Characterization and applications of a bismuth bulk electrode. Electroanalysis: An International Journal Devoted to Fundamental and Practical Aspects of Electroanalysis.

[45] Chen K., Wei X., Ding J., Wang W., Lu J. (2022). Bi-Sn-In phase change material with low melting point and high cyclic stability for thermal energy storage and management. Chem. Eng. J..

[46] He Y., Zhao Y., Fan L., Wang X., Duan M., Wang H., Zhu X., Liu J. (2021). Injectable affinity and remote magnetothermal effects of bi-based alloy for long-term bone defect repair and analgesia. Adv. Sci..

[47] Fu J., Gao J., Chen S., Qin P., Shi J., Liu J. (2019). Self-encapsulation liquid metal materials for flexible and stretchable electrical conductors. Royal Society of Chemistry Advances.

[48] Zhang R., Ye Z., Gao M., Gao C., Zhang X., Li L., Gui L. (2020). Liquid metal electrode-enabled flexible microdroplet sensor. Lab Chip.

[49] Yu L., Pereira A.L.C., Tran D.N.H., Santos A.M.C., Losic D. (2021). Bismuth Oxide Films for X-ray shielding: Effects of particle size and structural morphology. Mater. Chem. Phys..

[50] Maestre C.V., Santos G.N. (2023). Effect of bismuth oxide nanoparticle on the electromagnetic interference shielding and thermal stability of industrial waste based-geopolymer composites. Sci. Rep..

[51] Saleh A., Abdelhakim N.A. (2025). Synthesis, physical, structure, mechanical and ionizing radiation shielding properties of some bismuth-based alloys: Comparative investigation. Radiat. Phys. Chem..

[52] Kaewpirom S., Chousangsuntorn K., Boonsang S. (2022). Evaluation of micro-and nano-bismuth (III) oxide coated fabric for environmentally friendly X-ray shielding materials. ACS Omega.

[53] Tishkevich D., Grabchikov S., Lastovskii S., Trukhanov S., Zubar T., Vasin D., Trukhanov A. (2018). Correlation of the synthesis conditions and microstructure for Bi-based electron shields production. J. Alloys Compd..

[54] Roy C.K., Bhavnani S., Hamilton M.C., Johnson R.W., Knight R.W., Harris D.K. (2016). Thermal performance of low melting temperature alloys at the interface between dissimilar materials. Appl. Therm. Eng..

[55] Deng Y., E E., Li J., Jiang Y., Mei S., Yu Y. (2023). Materials, fundamentals, and technologies of liquid metals toward carbon neutrality. Sci. China Technol. Sci..

[56] Bie B., Xu W., Lv Y. (2023). Liquid metal-based textiles for smart clothes. Sci. China Technol. Sci..

[57] Hodge J., Quint C. (2019). The improvement of cell infiltration in an electrospun scaffold with multiple synthetic biodegradable polymers using sacrificial PEO microparticles. J. Biomed. Mater. Res..

[58] Mises R.v. (1913). Mechanik der festen Körper im plastisch-deformablen Zustand. Nachrichten von der Gesellschaft der Wissenschaften zu Göttingen. Mathematisch-Physikalische Klasse.

[59] Tang S., Cheng S., Yu C., Wu Z., Tan Y., Zeng X. (2025). Impedance-matchable 3D MXene sponge/NiFe@NC heterostructure with tunable pores for efficient electromagnetic wave absorption and thermal resistance. Sci. China Mater..

[60] Zhan B., Hao Y., Qi X., Qu Y., Ding J., Yang J., Gong X., Chen Y., Peng Q., Zhong W. (2023). Multifunctional cellular carbon foams derived from chitosan toward self-cleaning, thermal insulation, and highly efficient microwave absorption properties. Nano Res..

[61] Mei J., Luo J., Zhao T., Jiang S., Wu Y., Dai Z., Xie Y. (2025). Morphology engineering of MIL-88A-derived 0D/1D/2D nanocomposites toward wideband microwave absorption. J. Mater. Sci. Technol..

[62] Liu J., Tan S., Yang X., Zhu J., Yan X., Chen T., Ji G. (2026). A multi-scale cross-band defense system integrating decoupled visible, dynamic infrared camouflage and electromagnetic shielding. Nano-Micro Lett..

[63] Zhu W., Feng R., Yang W., Li Z., Zhang C., Du S., Cao Z., Dong J., Kong L., Li Y. (2025). Robust and highly conductive graphene-based foams for efficient thermal insulation, flame retardancy and electromagnetic interference shielding. Adv. Funct. Mater..

[64] Zhu W., Feng R., Li Z., Yang W., Zhang C., Du S., Ding Z., Cao Z., Dong J., Kong L., Li Y. (2025). Design strategies and research progress of macrostructure graphene-based electromagnetic shielding materials. Carbon.

[65] Huang J., Zeng X., Jiang X., Deng X., Wu Z., Gao Y. (2025). In situ construction of MXene derivatives and rare metal doping in nanofibers for multifunctional and ultrathin electromagnetic responses. Adv. Funct. Mater..

[66] Zeng X., Deng X., Yu Z., Zhang X., Lu J., Gao Y. (2026). Evolution of Fe single atom in SiOC ceramic fibers and their high-temperature and ultrathin electromagnetic wave absorption. Adv. Mater..

[67] Yan X., Guo F., Lin Y., Ji G. (2025). Recent advances in multifunctional electromagnetic interference shielding materials. Chemical Communications (Cambridge, England).

[68] Li Z., Yang W., Zhang C., Ding Z., Du S., Wu X., Dong J., Zhu W., Liu S., Lin Y. (2025). Multiscale structural design of heterostructured carbon/boron nitride aerogels for efficient thermal conductivity and broadband electromagnetic wave absorption. Chem. Eng. J..

[69] Yang W., Bai H., Jiang B., Wang C., Ye W., Li Z., Xu C., Wang X., Li Y. (2022). Flexible and densified graphene/waterborne polyurethane composite film with thermal conducting property for high performance electromagnetic interference shielding. Nano Res..

[70] Feng R., Zhu W., Yang W., Li S., Zhang C., Li Z., Du S., Li Y. (2025). Scalable production of flexible and multifunctional graphene-based polymer composite film for high-performance electromagnetic interference shielding. Carbon.

[71] Song W., Cao M., Lu M., Bi S., Wang C., Liu J., Yuan J., Fan L. (2014). Flexible graphene/polymer composite films in sandwich structures for effective electromagnetic interference shielding. Carbon.

[72] Otasowie P.O., Ogujor E.A. (2009). Voltage standing wave ratio measurement and prediction. International of Physical Sciences.

[73] Lendlein A., Gould O.E.C. (2019). Reprogrammable recovery and actuation behaviour of shape-memory polymers. Nat. Rev. Mater..

[74] Wang C., Guo Y., Chen J., Zhu Y. (2023). Transparent and flexible electromagnetic interference shielding film based on Ag nanowires/ionic liquids/thermoplastic polyurethane ternary composites. Compos. Commun..

[75] Kalia R., Chauhan A., Jasrotia R., Ijaz M.F., Batoo K.M., Verma R. (2025). Bi-doped cobalt ferrite nanoparticles for electromagnetic interference (EMI) shielding applications. J. Mater. Sci. Mater. Electron..

[76] Harshapriya P., Basandrai D., Kaur P. (2023). Structural, magnetic, microwave absorption and electromagnetic properties of Y-Ag-doped bismuth ferrite nanoparticles for commercial applications. Appl. Phys. Mater. Sci. Process.

[77] Harshapriya P., Kaur P., Basandrai D. (2025). Fabrication and characterization of BFO/BTO/TiO2 hybrid nanocomposites as an EMI shielding material in X-band for commercial applications. Mater. Sci. Eng., B.

[78] Li Y., Cao W., Yuan J., Wang D., Cao M. (2015). Nd doping of bismuth ferrite to tune electromagnetic properties and increase microwave absorption by magnetic–dielectric synergy. J. Mater. Chem. C.

