# Supplementary material for: Porous bismuth-based liquid metal as multifunctional material
Source: iScience. 2026 May 22;29(6):116028. doi: 10.1016/j.isci.2026.116028 (PMC13218255; doi:10.1016/j.isci.2026.116028)
Supplement: Document S1. Figures S1–S8 and Table S1 [file mmc1.pdf]

iScience, Volume *29*

Supplemental information

Porous bismuth-based liquid metal

as multifunctional material

Ju Wang, Yan Wang, Yunlong Bai, Jingyi Li, Jie Zhang, Minghui Guo, Zhongshan Deng, Yong Zhang, Jinpeng Zhang, Wei Rao, and Jing Liu

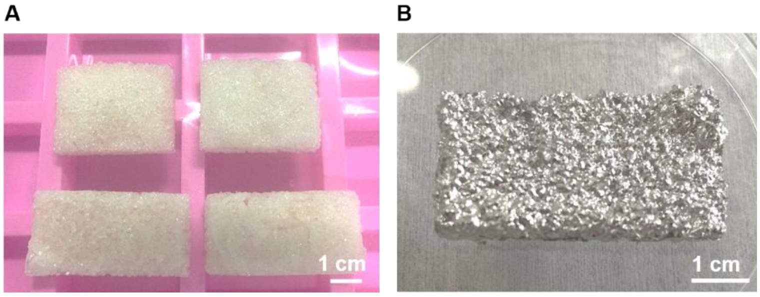

Figure S1. Photographs of preparing PBLM via the sugar sacrificial template method, related to Figure 1. (A) Sugar template obtained after freeze drying. (B) Composite after infiltration and wetting of the sugar template by liquid metal. Scale bars: 1 cm.

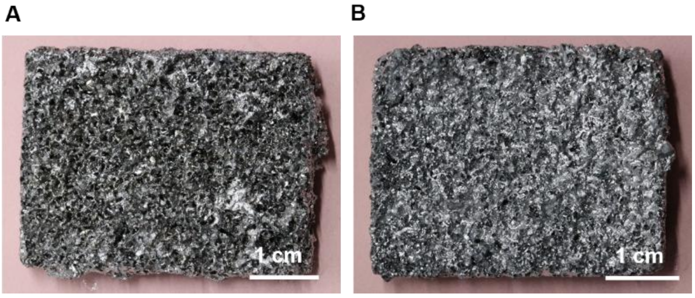

Figure S2. Surface morphology and pore distribution characteristics of PBLM, related to Figure 2. (A) The sample prepared using the lower mesh template (#20) exhibited larger pore sizes and more prominent pore features. (B) The sample prepared using the higher mesh template (#25) exhibited smaller pores and a denser pore distribution. Scale bars: 1 cm.

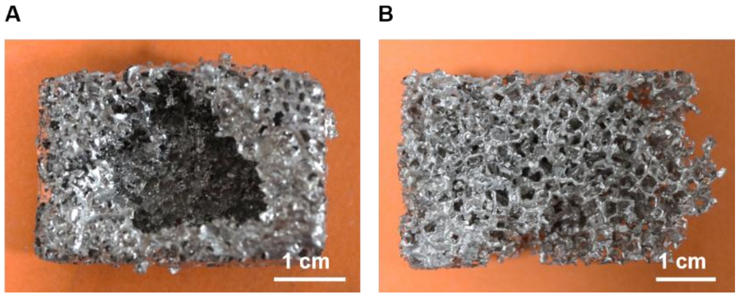

Figure S3. Representative morphologies of PBLM prepared using additional template particle sizes, related to Figure 3. (A) The #30 sample showed severe structural defects. (B) The #10 sample showed increased fragility. Scale bars: 1 cm.

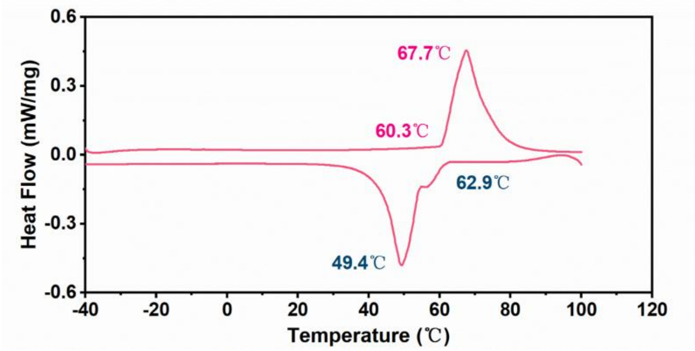

Figure S4. DSC test of PBLM, related to Figure 3. The sample was prepared using the #20 sieve derived sugar template.

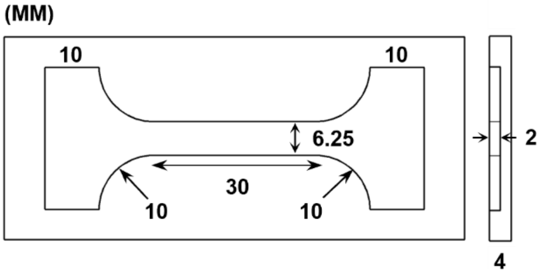

Figure S5. Schematic of specimen dimensions for mechanical testing, related to Figure 3.

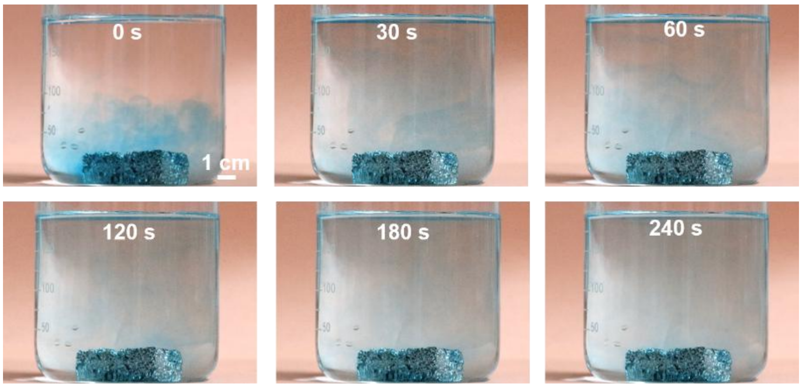

Figure S6. Time-dependent release of dyed liquid in the vertical direction, related to Figure 5. Scale bars: 1 cm.

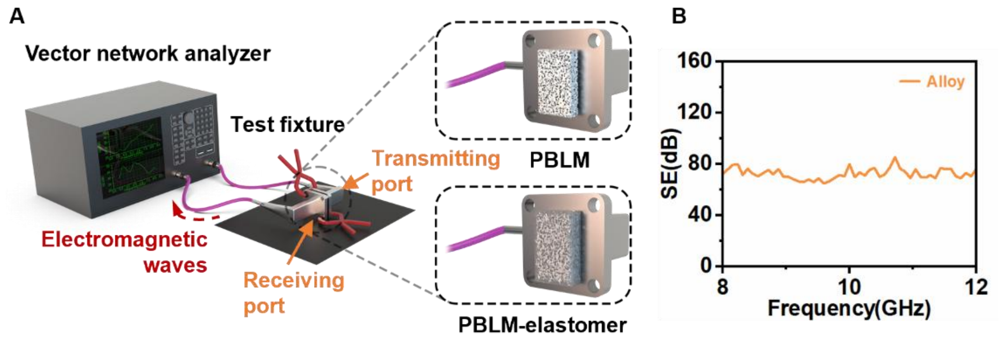

Figure S7. Electromagnetic shielding measurement setup and shielding effectiveness of the dense alloy, related to Figure 6. (A) Schematic illustration of the electromagnetic shielding measurement setup. (B) Shielding effectiveness of dense $Bi_{31.6}In_{48.8}Sn_{19.6}$ alloy in the X band.

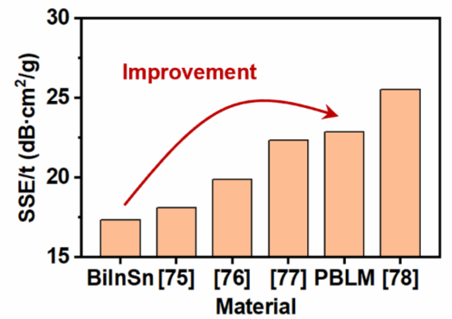

Figure S8. Comparison of the absolute shielding effectiveness (SSE/t) of PBLM, $Bi_{31.6}In_{48.8}Sn_{19.6}$ alloy, and representative shielding materials reported in the literature, related to Figure 8.

Table S1. Basic physical properties of typical liquid metal compositions[S1-10], related to Figure 1.

Liquid metal type	Melting point °C	Density g/cm^3	Specific heat capacity J/g/K	Conductivity 10^6S/m	Thermal conductivity W/m/K
Hg	-38.8	13.564	0.139	-	8.34
Ga	29.8	5.907[a]	0.37	3.7[a]	40.6[a] /29.4[b]
In	156.6	7.31[a]	0.23	11.95[a]	36.4[c]
Sn	231.9	5.75[a]	0.221	8.69[d]	15.08[e]
Bi	271.32	9.75[a]	0.12	0.95	7.87
$Na_{22.2}K_{77.8}$	-12	0.75	-	-	26.2
$Ga_{75.5}In_{24.5}$	15.7	6.28[a]	-	3.4[a]	27.5
$Bi_{31.6}In_{48.8}Sn_{19.6}$	60.2	8.043[a]	0.297	-	14.5[f]

Note: [a]：Around room temperature；[b]：50 °C; [c]：160 °C; [d]：0 °C; [e]：77 °C; [f]：80 °C.

Original code for the sphere distribution model used in finite element simulation

```
main
    clear
    X1=0;Y1=0;Z1=0;
    X2=5;Y2=5;Z2=5;
    Fai=0.45;
    Vmax=(X2-X1)*(Y2-Y1)*(Z2-Z1)*(1-Fai);
    New_Sugar(X1, Y1, Z1, X2, Y2, Z2, Vmax);
function New_Sugar(X1, Y1, Z1, X2, Y2, Z2, Vmax)
    close all
    MAX_ITER = 1000000;
    i = 1;
    s = [];
    V = 0;
    rng('shuffle');
    max_balls = 30000;
    s = NaN(4, max_balls);
    h = waitbar(0, 'Program starting...');
    kdtree = KDTreeSearcher([]);
    tic;
    while V < Vmax && i < MAX_ITER
        radii = 0.75 / 2;
        for tempR = radii
            temp_s = [X1 + (X2 - X1) * rand(); ...
                      Y1 + (Y2 - Y1) * rand(); ...
                      Z1 + (Z2 - Z1) * rand(); ...
                      tempR];
            queryPoint = temp_s(1:3, :)';
            if i > 1
                neighbors = rangesearch(kdtree, queryPoint, 0.85);
            else
                neighbors = [];
            end
            is_valid = true;
            if ~isempty(neighbors) && ~isempty(neighbors{1})
                for l = 1:length(neighbors{1})
                    neighbor_idx = neighbors{1}(l);
                    dis = sum((temp_s(1:3) - s(1:3, neighbor_idx)).^2);
                    sum_radius = tempR + s(4, neighbor_idx);
                    if dis < sum_radius^2
                        if dis < 0.85^2 && dis > 0.8^2
                        else
                            is_valid = false;
```

```
                    break;
                end
            end
        end
    end
    if is_valid
        s(:, i) = temp_s;
        V = V + (4 / 3) * pi * tempR^3;
        i = i + 1;
        found = true;
        if i > max_balls
            s = [s, NaN(4, max_balls)];
            max_balls = max_balls * 2;
        end
        kdtree = KDTreeSearcher(s(1:3, 1:i-1)');
        waitbar(V / Vmax, h, sprintf('Progress: %.2f%%, Number of spheres: %d', V
/ Vmax * 100, i - 1));
        break;
    end
end
if V >= Vmax
    break;
end
end
close(h);
fprintf('Generation completed, elapsed time: %.2f seconds\n', toc);
X = s(1, 1:i-1);
Y = s(2, 1:i-1);
Z = s(3, 1:i-1);
R = s(4, 1:i-1);
save('data.mat', 'X', 'Y', 'Z', 'R');
figure;
[x, y, z] = sphere(20);
for t = 1:i-1
    surf(R(t) * x + X(t), R(t) * y + Y(t), R(t) * z + Z(t), 'EdgeColor', 'none', 'FaceAlpha',
0.7);
    hold on;
end
axis equal;
grid on;
xlabel('X');
ylabel('Y');
zlabel('Z');
```

```
    title('Sphere distribution');
end
```

REFERENCES

[S1] Song, J., Lui, T., Chang, Y., and Chen, L. (2005). Compositional effects on the microstructure and vibration fracture properties of Sn–Zn–Bi alloys. Journal of Alloys and Compounds *403*, 191–196. 10.1016/j.jallcom.2005.05.016.

[S2] Gao, J., Chen, S., Liu, T., Ye, J., and Liu, J. (2021). Additive manufacture of low melting point metal porous materials: Capabilities, potential applications and challenges. Materials Today *49*, 201–230. 10.1016/j.mattod.2021.03.019.

[S3] Zhang, Q., Yao, Y., Gao, J., Yang, X., Zhang, P., Deng, Z., and Liu, J. (2020). Thermal evaluation of the injectable liquid metal bone cement in orthopedic treatment. Science China Technological Sciences *63*, 446–458. 10.1007/s11431-018-9446-6.

[S4] Shan, W., Lu, T., and Majidi, C. (2013). Soft-matter composites with electrically tunable elastic rigidity. Smart Materials and Structures *22*, 085005. 10.1088/0964-1726/22/8/085005.

[S5] Liu, J., Sheng, L., and He, Z. (2018). Liquid metal soft machines: principles and applications (Springer). 10.1007/978-981-13-2709-4.

[S6] Gao, J., Ye, J., Chen, S., Gong, J., Wang, Q., and Liu, J. (2021). Liquid metal foaming via decomposition agents. ACS Applied Materials & Interfaces *13*, 17093–17103. 10.1021/acsami.1c01731.

[S7] Zhang, X., and Liu, J. (2020). Perspective on liquid metal enabled space science and technology. Science China Technological Sciences *63*, 1127–1140. 10.1007/s11431-019-1534-7.

[S8] Zhu, L., Wang, B., Handschuh-Wang, S., and Zhou, X. (2020). Liquid metal–based soft microfluidics. Small *16*, 1903841. 10.1002/smll.201903841.

[S9] Ge, H., Li, H., Mei, S., and Liu, J. (2013). Low melting point liquid metal as a new class of phase change material: An emerging frontier in energy area. Renewable and Sustainable Energy Reviews *21*, 331–346. 10.1016/j.rser.2013.01.008.

[S10] Yang, X., and Liu, J. (2018). Advances in liquid metal science and technology in chip cooling and thermal management. In Advances in Heat Transfer, (Elsevier), pp. 187–300. 10.1016/bs.aiht.2018.07.002.
